# Properties, Applications and Recent Developments of Cellular Solid Materials: A Review

**DOI:** 10.3390/ma16227076

**Published:** 2023-11-08

**Authors:** Girolamo Costanza, Dinesh Solaiyappan, Maria Elisa Tata

**Affiliations:** Industrial Engineering Department, University of Rome Tor Vergata, Via del Politecnico 1, 00133 Rome, Italy; dineshsolaiappan95@gmail.com (D.S.); elisa.tata@uniroma2.it (M.E.T.)

**Keywords:** cellular solids, sandwich materials, mechanical characterization, honeycomb and foam materials, physical properties, industrial applications

## Abstract

Cellular solids are materials made up of cells with solid edges or faces that are piled together to fit a certain space. These materials are already present in nature and have already been utilized in the past. Some examples are wood, cork, sponge and coral. New cellular solids replicating natural ones have been manufactured, such as honeycomb materials and foams, which have a variety of applications because of their special characteristics such as being lightweight, insulation, cushioning and energy absorption derived from the cellular structure. Cellular solids have interesting thermal, physical and mechanical properties in comparison with bulk solids: density, thermal conductivity, Young’s modulus and compressive strength. This huge extension of properties allows for applications that cannot easily be extended to fully dense solids and offers enormous potential for engineering creativity. Their Low densities allow lightweight and rigid components to be designed, such as sandwich panels and large portable and floating structures of all types. Their low thermal conductivity enables cheap and reliable thermal insulation, which can only be improved by expensive vacuum-based methods. Their low stiffness makes the foams ideal for a wide range of applications, such as shock absorbers. Low strengths and large compressive strains make the foams attractive for energy-absorbing applications. In this work, their main properties, applications (real and potential) and recent developments are presented, summarized and discussed.

## 1. Introduction

**Figure 1:**
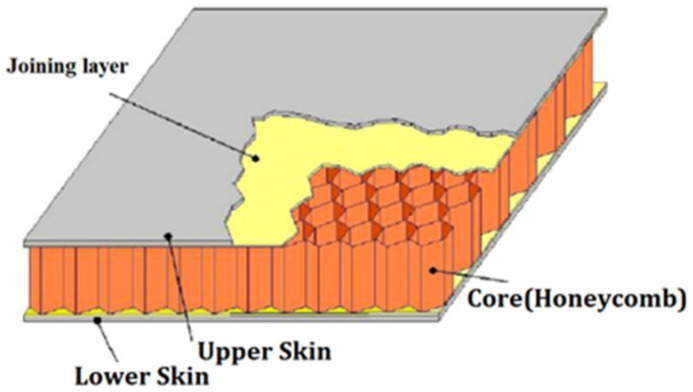
Typical cellular honeycomb sandwich structure [6].

Many industries, including the automotive, aerospace, sport and biomedical sectors, have a critical need for materials that are extremely reliable (fatigue tolerant), stiff, performing and lightweight [1]. Cellular solids are a class of materials made up of cells with solid edges or faces that are piled together to fit a certain space. These materials are present in nature and have been utilized by people for ages. Some examples are wood, cork, sponge and coral. Recently, new cellular solids, inspired by natural ones, have been manufactured such as honeycomb materials and polymer foams, which have a variety of uses because of their special characteristics. Lightweight, insulating, cushioning and energy-absorbing are characteristics that come from having a cellular structure. Cellular solids are frequently disregarded despite their significance as an engineering material. Yet, they are commercially significant and widely manufactured and consumed. There is a high demand for porous biomaterials that imitate the varied characteristics of bone [2]. This is because they are useful for replacing bone and used in different kinds of orthopedic implants that must minimize stress shielding while providing adequate mechanical support and a long fatigue life. Additionally, the permeability of bone-like porous biomaterials must be suitably constructed to enable for the nutrient and oxygen delivery of cells dwelling in the interior of the porous biomaterials. When compared to other types of biomaterials, fully porous biomaterials offer several benefits [2]. In-plane qualities can change greatly with different loading situations; however, many researches focus on out-of-plane mechanical properties, making investigations into various loading scenarios inherently outside the study’s purview. Isotropy is a crucial factor to consider for applications where the directions of forces cannot be assumed, such as lightweight sandwich panels in aviation and explosion protection in military vehicles. Some honeycombs, including those with a hexagonal foundation and those presumed to be totally isotropic, may be anisotropic for large deformations while remaining isotropic for tiny deformations [3]. Polymeric foams are frequently utilized for a variety of purposes, including energy absorption, noise reduction, thermal insulation and floral design. The modeling of a substantial displacement, including the unloading component, is crucial for an effective foam design in several engineering applications, such as energy absorption. Over the past ten years, several foam deformation models have been developed [4]. More than 60% of this market is made up of cellular materials, which are used in the building and construction, automotive, bedding, furniture and insulation industries. There are two primary varieties of foam that may be produced: flexible with open cells that have stress and tensile qualities, such as those found in furniture or bedding, and stiff with closed cells that have low thermal conductivity, low density and good dimensional stability, primarily for thermal insulation, such as those found in construction industries [5]. The microstructure or morphology of these cellular materials is greatly influenced by the formulation stage, which also affects the final foam qualities. Commercial PU cellular materials are now mostly based on fossil resources, notwithstanding the possibility of using certain partly biobased components (polyols). But to meet social expectations, new materials will combine excellent performance with little environmental effect. These results in distinct renewable macromolecular designs as novel biobased substances fusing diverse domains such as biotech, chemistry, science and materials engineering are increasingly utilized in complicated formulations for renewable foams. Producing biodegradable polymers from fossil resources like polycaprolactone is another option. The huge and growing issue of plastic dispersion and accumulation in the environment is related to the development of biodegradable polymers. Designing sustainable polymeric materials is a must given the real buildup of microplastics in the ocean and global warming. Sandwich structures are a specific type of laminated composite made of a lightweight core (such as honeycomb, truss or foam) and two stiff facings at the top and bottom made of fiber-reinforced polymer (FRP) composites, epoxy/carbon composites or other metal alloys. Using an appropriate joining method, the face sheet and the core were joined together. An illustration of a typical cellular honeycomb sandwich structure is shown in Figure 1 [6].

## 2. Sandwich Composite Cellular Materials

**Figure 2:**
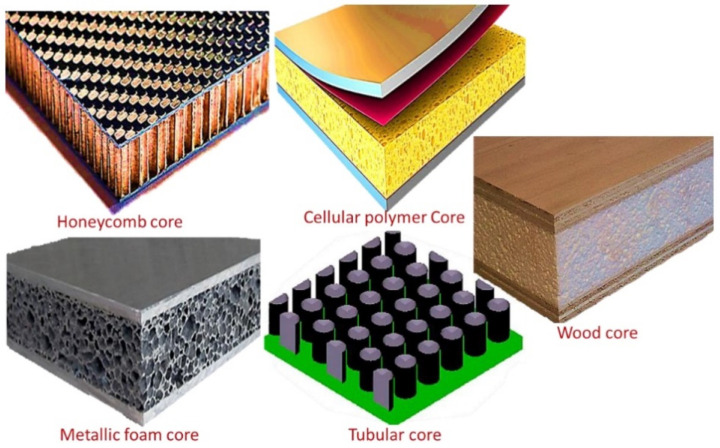
Different types of sandwich composite structures [7].

Sandwich composite cellular materials utilized in building and manufacturing are arranged to combine good quality and robustness with low weight. They are created by sandwiching a lightweight center texture, such as foam or honeycomb, between two outside layers of solidified, strong materials, such as carbon fiber or fiberglass. The most recently developed sandwich structure gives astonishing strength-to-weight and stiffness-to-weight ratios, making it a well-known choice in applications where weight may be an essential consideration, such as flying, car, marine and advancement businesses. The points of interest in sandwich composite materials over routine materials are that they incorporate higher quality and solidity, a prevalent resistance to influence and advanced separator properties. Moreover, sandwich composites can be molded into complex shapes, diminishing the need for additional components and facilitating the manufacturing plan. Sandwich composite materials have a wide range of applications, from aviation and defense to renewable imperatives and systems. They are utilized to create everything from plane and carrying components to wind turbine edges, vessel outlines and building sheets. Figure 2 shows different designs of sandwich composite structures [7].

### 2.1. Honeycomb Core

**Figure 3:**
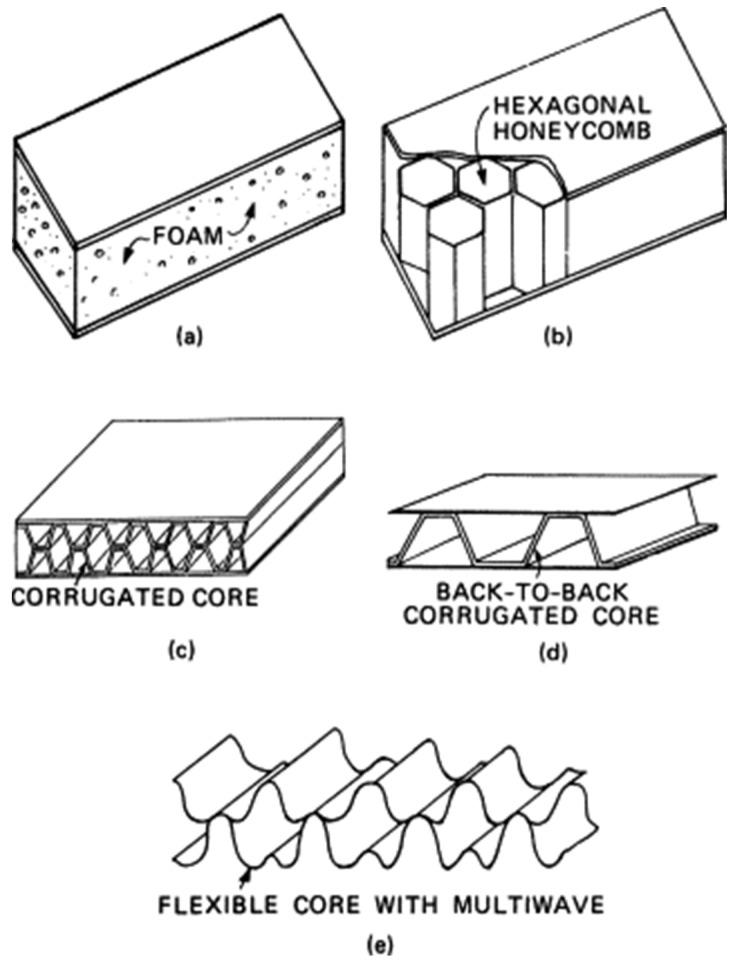
Types of honeycomb core materials in sandwich composites: (**a**) foam, (**b**) hexagonal honeycomb, (**c**) corrugated, (**d**) back-to-back corrugated and (**e**) flexible core with multiwave [8].

After defining a sandwich composite, it is possible to categorize different types of sandwich structures based on the materials used for the core and the front [8]. For instance, Figure 3a depicts a sandwich composite with a foam-type core, Figure 3b depicts a hexagonal honeycomb, Figure 3c depicts a unidirectional corrugated core, and Figure 3d depicts a back-to-back corrugation variation of the unidirectional corrugated core. Two fluted metal sheets are connected to the corrugated core in this instance. Typically, aluminum foil is used to make the honeycomb-shaped core. The typical honeycomb core has a propensity to bend anticlockwise and is difficult to fit into a cylindrical surface. The flexible core with multiwave, an enhanced variant, appears to offer remarkable formability into compound curvatures [8]. Additionally, it offers greater shear strengths than a comparable hexagonal core with the same density. To create new core shape combinations, a superplastic forming approach has been used. The initial outcomes with this novel technique appear to be quite encouraging. The main advantages of a honeycomb core are toughness, lightness, recyclability, cost-effectiveness and customizability.

Aluminum, stainless steel or other alloys are all acceptable materials for facing sheets. As face sheet materials, fiber-reinforced laminates such as glass–epoxy, graphite–epoxy and boron–epoxy have often been employed. On the other hand, plywood, glass-reinforced cement, plasterboard, hardboard and other materials are often utilized in the construction of buildings as facings [8]. Almost any form of core may be bonded together with any type of face sheet depending on how the sandwich composite will be used. Sandwich composites may be produced for specific purposes, but naturally, commercially available composites are significantly less expensive than materials that are built to order.

### 2.2. Cellular Polymer Core

Due to their heat sensitivity and viscoelasticity, polymers are quickly melted or subject to thermal rupture. In general, micro-flaws and crazing in highly stress-concentrated places signal the start of fatigue breakdown in polymeric materials. Internal and exterior surface imperfections, voids and poorly bonded matrix interfacial regions, which have a significant impact on the mechanical strength and subsequently lead to deformation, are the main causes of crazing [9]. Up until the critical fracture size is achieved, which results in an abrupt catastrophic failure, continued cyclic loading produces plastic deformation and crack propagation in polymers. Analyzing the beginning and growth of fractures allows for a detailed study of polymeric material fractures under fatigue. Due to their special qualities and myriad prospective uses in the automotive, aerospace, building, medicinal and electrical sectors, cellular polymer cores have opened a new frontier in material research [10]. Few technological fields need multidisciplinary collaboration more or have a greater influence on the quality and longevity of life than polymer cores; as a result, researchers in the fields of materials, biology and engineering sciences, among others, face enormous hurdles [11].

### 2.3. Wood Core

**Figure 4:**
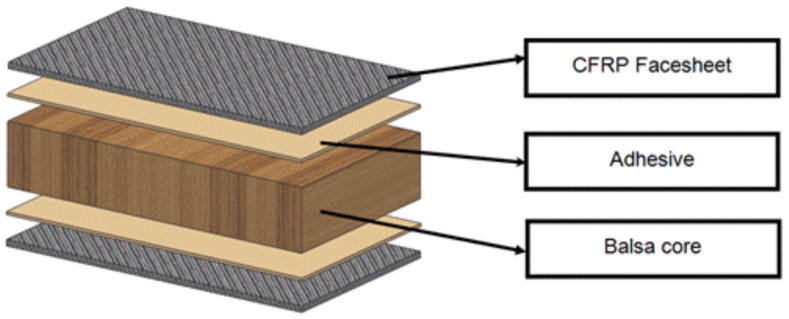
The balsa wood core sandwich structure [12].

Sandwich constructions made of a balsa wood core and a carbon-fiber-reinforced polymer (CFRP) skin joined by an adhesive film are examined in [12]. Due to the benefits they offer, sandwich constructions with a balsa wood core are often utilized in the rail, road, building, renewable energy and aerospace sectors. A thorough investigation of the compression response and a failure analysis of samples made from readily available balsa wood that were tested in several directions (axial, radial and tangential) were given [12]. The balsa wood demonstrated a linear rise in the modulus of elasticity and strength vs. density when exposed to axial compression, flexure and torsion. Figure 4 shows the wood core of the balsa wood sandwich structure [12]. Balsa’s air-dry density was largely responsible for its mechanical characteristics. The end-grain balsa panels from Ecuador’s shear characteristics show the influence of the density, adhesive connections and shear plane on the shear characteristics of the balsa panels. The balsa’s density grew along with its shear stiffness and strength [13]. Due to plastic deformations in the tracheid, certain specimens showed notable ductility.

### 2.4. Metallic Foam Core

Metallic foams have generated a lot of interest, in part because of improvements in processing that have made the material more affordable and in part because they can exhibit appealing property combinations, notably in terms of specific stiffness and specific energy absorption. Metallic foams may be produced using a variety of techniques. Some of them entail treating the metal in the liquid or semisolid state, especially those that produce closed-cell structures [14]. Open-cell metallic foams are less rigid than closed-cell metallic foams, but since they allow fluids to flow easily through heated structures, they offer advantages that may be used in heat transfer and multifunctional load-supporting applications [15,16]. They can be utilized as high-temperature supports for catalysts and electrodes in electrochemical cells due to their high surface-area-to-volume ratio. Open-cell metal foams can be created through electrolytic deposition, chemical or physical vapor deposition on polymer templates, investment casting utilizing an open-cell mold and the PM method [15].

**Figure 5:**
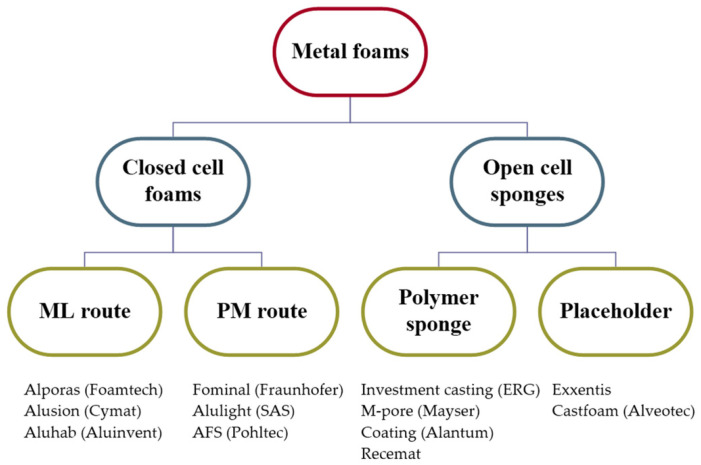
Schematic grouping of the economically most significant metal foam and sponge production techniques, together with some illustrative brand names and industries [17].

For foams, sponges and porous materials with macroscopic pores, there are several production techniques. Only the commercially most pertinent approaches will be discussed here. The two primary families of closed-cell foams, also known as the production routes, are the ML route and the powder metallurgical (PM) approach. The direct and indirect foaming procedures are other names for them. In the literature, most of these manufacturing processes and their variants have previously been covered [17]. There are other production processes as well, particularly when it comes to metallic sponges, which cannot be immediately foamed since the gas would leak. Figure 5 illustrates the schematic grouping of the economically most significant metal foam and sponge production techniques, together with some illustrative brand names and industries [17]. They do not directly contribute to foaming in the traditional meaning of the word, but they do result in a structure that resembles foam. These techniques may also be separated into two primary groups: the first uses a polymeric sponge structure as a pattern or carrier, while the second uses a removable placeholder. Typically, but not always, their applications are centered on their functionality.

### 2.5. Tubular Core

Due to their capacity to sustain transverse loads with no weight penalty and absorb significant amounts of plastic energy, sandwich-type cladding structures with lightweight cores are currently becoming more attractive for blast mitigation applications, including armor systems [18]. The performance of the sandwich panels is significantly influenced by the sandwich core selection. Typical core materials include wood, various foams and tubular constructions. Tubular constructions are frequently employed due to their great energy absorption capacities. Sandwich panels use tubular constructions as cores under blast and dynamic stresses [18].

**Figure 6:**
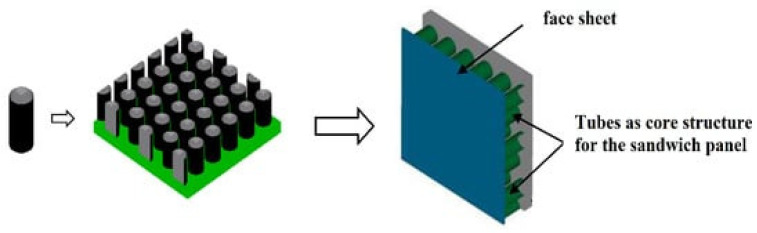
General example of tubular core sandwich structure [19].

The basic component of this sandwich construction is made up of tubes. Applications for low-velocity impacts (perforation), shock waves and crashworthiness are the most common ones of tubular cores. Figure 6 illustrates an example of a tubular core sandwich structure [19]. Metals (aluminum/stainless steel) or fiber composites are frequently employed as sandwich construction face sheets. Metals and polymers were the two most often employed materials for the core construction [19]. The following are some of the major conclusions: Although less perforation-related, such a core design offers strong blast protection and crashworthiness. Because it influences the creation of the plastic hinge, the tube configuration between the face sheets is essential; foam-filled tubes provide strong energy absorption properties.

## 3. Manufacturing Process of Cellular Solids Sandwich Structures

**Figure 7:**
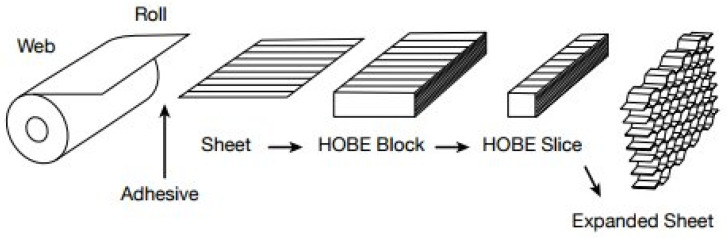
Manufacturing process for corrugated honeycomb cores [20].

The cellular solids’ sandwich structure manufacturing process includes skin manufacturing, honeycomb core preparation, adhesive application, sandwich structure assembly, curing and finishing. This process ensures the production of lightweight, high-strength and rigid composite panels that are suitable for a variety of applications in industries such as aerospace, automotive and construction. Using a corrugated roller to create a corrugated sheet from a roll of aluminum foil is an alternate method. This sheet’s flat parts are coated with adhesive before sections of the corrugated sheets are placed on top of them, with the flats holding them together while the glue cures [20]. This is usually carried out for honeycomb cores that are not generated through the expansion process and have smaller cells. Figure 7 illustrates the manufacturing process for corrugated honeycomb cores [20]. Both techniques require gluing two sheets of aluminum foil together to form a honeycomb structure where the cell walls along one orientation have twice the wall thickness as compared to those in either oblique orientation. The “ribbon direction” is the name given to this position.

Sandwich panels are created by using an adhesive to join the face sheets to the core material [19]. This might happen at the same time as the composite material’s curing process in the case of face sheets made of carbon-fiber-reinforced polymers (CFRP) or other composite materials.

### 3.1. Prepreg Lay-Up Process

**Figure 8:**
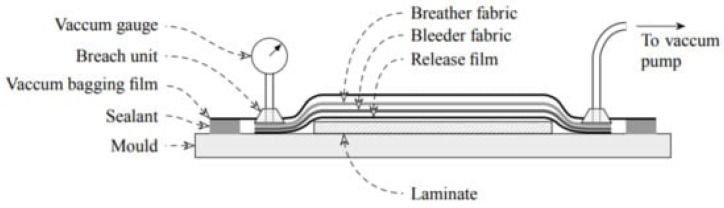
An overview of prepreg lay-up process [22].

The degassing lamination method is often used to create thermoset-based composite laminates. The prepreg lay-up process produces layers of the desired shape, which are assembled in this method in a certain orientation to create a laminate. Figure 8 shows an overview of the prepreg lay-up process [21]. Following layers of an absorbent substance (glass bleeder fabric), a fluorinated film to avoid adhering and, lastly, a vacuum bag, the laminate is coated. In an autoclave, the complete system is put on a flat metal tool surface, the bag is vacuumed and the temperature is raised steadily to encourage resin flow and polymerization [21]. This section will utilize the autoclave process as a case study to discuss how matrix properties affect high-performance composites during the process.

### 3.2. Hand Lay-Up Process

**Figure 9:**
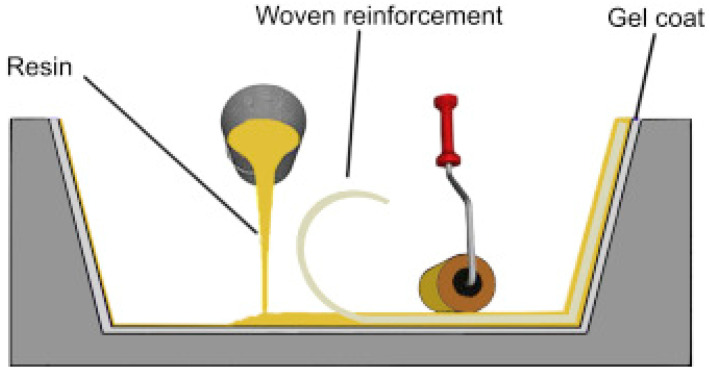
Hand lay-up process [23].

The earliest process for creating woven composites is called hand lay-up. To prevent a polymer from adhering to the mold surface, a released antiadhesive agent is applied first. The product’s surface is then made smooth by applying a thin plastic layer to the top and bottom of the mold plate. Figure 9 illustrates the hand lay-up process [23]. Cut into the necessary forms, the woven reinforcing layers are laid down on the mold’s surface. As a result, as previously stated, the resin combines with other materials and is evenly applied with the aid of an assist brush over the surface of the reinforcement that was already set up in the mold. The remaining mats are then positioned on top of the prior polymer layer, and pressure is applied with a roller to remove any trapped air bubbles as well as extra polymers [23]. To produce a single mat, the mold is then shut and the pressure is released. The woven composite is removed from the mold’s surface once it has finished curing at room temperature.

One of the oldest techniques employed in the sector is the hand lay-up method, often known as the wet lay-up method. Each ply is handled only by hand during the whole process, which entails the layer-by-layer stacking of the ply up to the necessary thickness [24]. Even though this approach is dependable, it is labor-intensive and takes longer to complete than modern production techniques. The employee’s expertise determines the quality. The complexity of the aircraft is a manufacturing constraint for this technology as well. Any materials, such as carbon or glass fiber, in any form (continuous fiber, chopped fiber, woven, etc.) are acceptable for this procedure [24].

### 3.3. Liquid Bonding

Using liquid adhesives or bonding agents to bind cellular or porous materials together is known as “liquid bonding” in cellular materials. To create a solid and long-lasting connection, cellular materials, such as foam, honeycomb structures or other porous substrates, frequently require specific bonding methods. The sandwich construction that will be produced will determine the ideal honeycomb core pressure range. For instance, thicker skins, lower vacuum bag pressure levels or greater internal core pressures may be necessary for cores with larger cell diameters to stabilize the core and minimize skin voids. The manufacturing process’s pressure sinks and sources determine how the ideal core pressure is reached [25]. Given the air permeability of the skin and the amount of vacuum bag pressure, the honeycomb core pressure will decrease. It will then rise because of a gas expansion brought on by rising temperatures and the presence of moisture or other volatiles. The foam cells have accessible routes to the atmospheric environment due to the micro-hole array, which entirely prevents Ostwald ripening and permits guided spontaneous evaporative formation of discretized liquid–air interfaces [26]. Instead, in a controlled manner, the micro-post array secures the grown interfaces at predetermined points. To illustrate the MNLP approach used to shape different liquid film networks and explain the mechanics of open-cell 2D liquid foam [26], it has been demonstrated that liquid-mediated materials may ultimately be constructed into long-lasting well-ordered micro-/nanostructure patterns by the evaporative thinning of the network to contain liquid-mediated or aqueous materials at the microscale and nanoscale.

### 3.4. Continuous Lamination

Continuous-fiber composites are laminated materials in which the orientation of the individual layers, plies or laminae enhances the strength in the main load direction. Unidirectional laminates are exceedingly stiff and robust when viewed in a parallel direction, but since the load must be borne by the much more fragile polymeric matrix, they are also quite weak when viewed in a perpendicular direction [27].

A conventional polymeric matrix typically has a tensile strength of only 5–10 ksi, but a high-strength fiber can have a tensile strength of 500 ksi or more. The matrix distributes the tension loads across the fibers and stabilizes and prevents the fibers from buckling under compression, while the fibers carry the longitudinal tension and compression loads. 

Laminated materials have long been used. The use of laminated glass-fiber-reinforced composites based on organic matrix resins in boat construction dates back more than 50 years, while applications for carbon fiber composite aircraft have gradually grown since the early 1970s [28]. Plywood is a conventional building material. For composite materials, whose mechanical behavior is strongly influenced by the manufacturing process, it has been acknowledged that the formulation of test standards is of utmost significance. Significant property alterations can occur because of changes in pressure, temperature or hygrometry.

### 3.5. Adhesive Bonding

**Figure 10:**
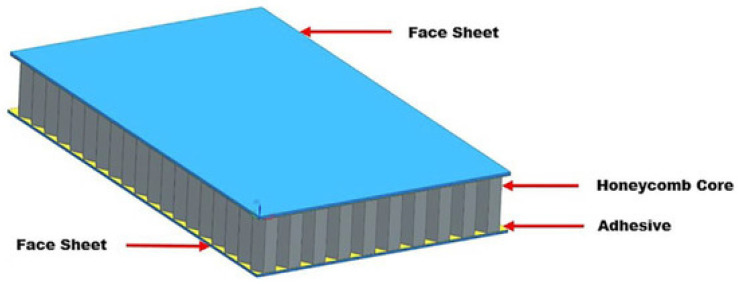
Adhesive bonding in honeycomb structure [29].

Humans have spent thousands of years learning about adhesives. Initially, materials derived from natural sources, such as birch tar, bituminous substances (asphalts) or animal collagen derivatives, were used, but the development of this technique has also been facilitated by the constant advancement of knowledge in the disciplines of chemistry, physics, materials engineering and mechanics [29]. There are already tens of thousands of different adhesive formulations on the market. There are adhesives made specifically for certain substrates, operational environments and joint loading scenarios, in addition to universal adhesives. There are adhesives for detachable joints, such as light paper adhesives and adhesives that are neutralized by solvents or heat, as well as adhesives for high-strength joints that call for specific stages in the bonding process (surface preparation, the application of the adhesive and the joint curing procedure). Figure 10 illustrates adhesive bonding in a honeycomb structure [29]. Today, a wide range of materials are bonded using this method, including simple building materials, tissues (adhesives used in biomedical engineering) and even the most intricately made honeycomb composites utilized in the aerospace sector. This demonstrates the current significance of the adhesive bonding process. Since adhesive bonding could be the only approach available for making joints in some circumstances, technology professionals are now adopting it more frequently than only as a complimentary alternative for attaching materials. Bolted connections, soldering and other conventional joining techniques are gradually being replaced by these joint types.

Fiber–metal laminates, sandwich structures and other joint assemblies that scale from the integration level of the part to the product employ adhesive bonding extensively. For instance, to achieve the synergistic benefits of both metal and composites, titanium alloys or steel alloys are adhesively linked to carbon-fiber-reinforced polymeric (CFRP) composite fan blades [30]. Additionally, metal alloys guard against erosion brought on by sand, stone or engine debris as well as damage from foreign object hits, such as those caused by bird strikes, hailstorms or rain. Damage causes include manufacturing flaws and internal residual stress release in use, which degrade the integrity of the composite blade throughout the course of its life and need “debonding”. To ensure appropriate adhesion, most structural adhesives create main chemical connections with the adherends, which are surface atoms that are either covalent or ionic.

## 4. Applications of Cellular Solid Structures

Due to their high stiffness-to-weight ratio, superior crash energy absorption, fire resistance, non-toxicity, low thermal conductivity, magnetic permeability and reduced density, cellular materials have the most promising applications and have been shown to be suitable for their applicability. In the case of cellular structures, there are additional application-specific advantages such as noise and energy absorption, mechanical dampening and filtration effects, in addition to significant weight reduction and material savings [31]. There are several materials available where weight reduction is the only factor to be considered; however, metal foams may be preferable if a low weight is also required, along with high energy absorption or heat resistance properties. Possible uses may be found in industries including light-weight building, crash energy absorption, noise reduction, transportation, construction, heat exchangers, purifiers, decorating and the arts, among others.

**Figure 11:**
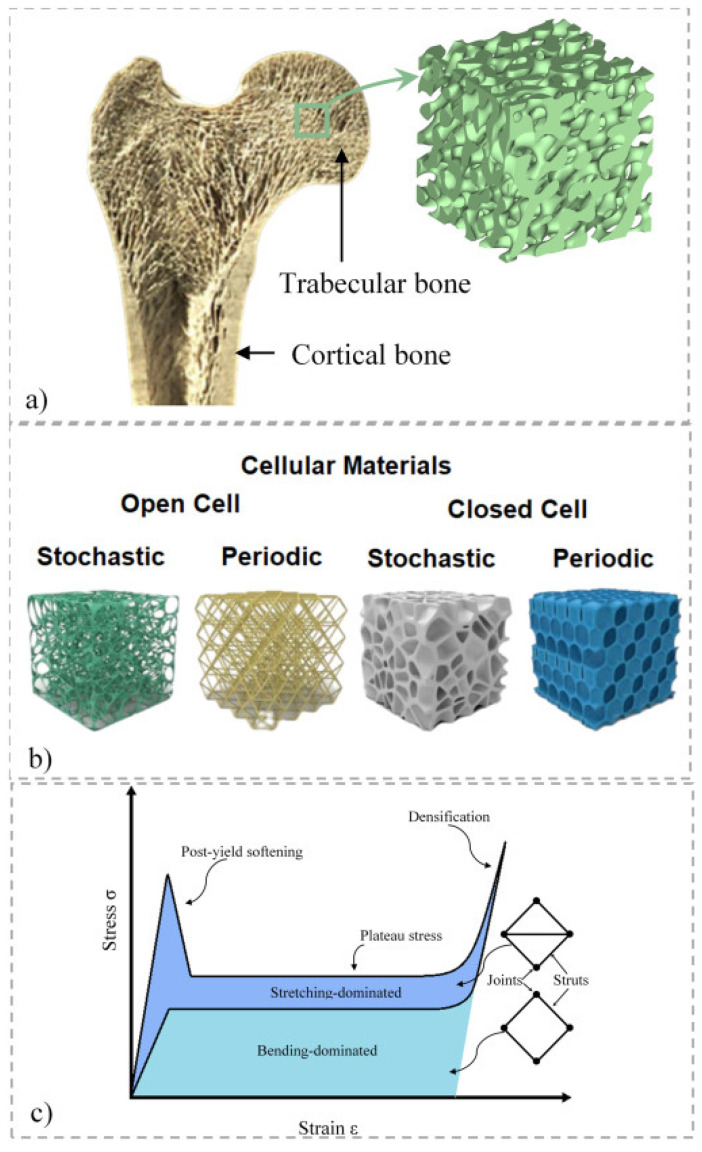
Cellular materials derived from nature. (**a**) The microstructure of bone; (**b**) the categorization of cellular materials; (**c**) the typical stress–strain response of cellular materials with bending and stretching as the dominant modes of deformation [32].

The solid and voided networks that make up the porous microstructure of cellular materials give them their distinctive properties. The growth of the subject of research known as cellular materials has been greatly impacted and supported by a variety of biological systems, including bone, honeycombs, marine sponges, wood and cork. One of the most fascinating natural biological materials for humans is likely bone (Figure 11) [32]. 

For support, protection and mobility, its microstructure has been developed to offer an unusual yet desired mix of strength and lightweight. Anatomically, bone is made up of cancellous (or trabecular) bone, which is largely porous, and cortical (i.e., compact) material, which is mostly solid. For instance, a thin cortical shell surrounds a porous cancellous core at the end of a femur bone, but a flat bone like the calvaria has a thin cancellous bone wedged between thick cortical shells. The Young’s modulus, strength, toughness and other mechanical characteristics of bone are governed by three basic factors: (1) the overall porosity of the bone, (2) the spatial distribution of the solid phase and (3) the degree of mineralization. Porosity is increased to save weight, but it also reduces strength and stiffness [32]. On the other hand, when the amount of mineralization rises, the bone strength rises as well. Additionally, solid matrices are stronger when orientated in that way because they offer more resistance to loading in that direction.

As a result, an optimum combination of these elements must be applied to the anatomical location of a bone and its intended use (external stress and functioning) to adapt the mechanical properties to acceptable and desired levels. The unique combination of strength, light weight and design flexibility of sandwich construction results in it having a wide range of applications in a variety of industries. They are commonly used in aerospace applications such as aerospace vehicles. Due to their mechanical, thermal, acoustic and electromagnetic properties, cellular materials are appealing for a variety of engineering applications, with the use of these materials for energy absorption and load attenuation seeing a steady increase in the building, aerospace, defense, transportation and biomedical sectors [33]. Because it is extremely light, less costly, fungus-resistant and impermeable, EPS could be a viable substitute for many of the primary materials now utilized in maritime applications. Unfortunately, it is also a somewhat weaker material, and because of this, it is prone to delamination and damage due to its poor shear and compressive strength. However, by thickening the core or utilizing shear webs, these restrictions can usually be circumvented. Lattice webs, a variation on the shear web idea, are currently being used in civil infrastructure applications. Unfortunately, polyester resin cannot be used with this material. However, this issue can usually be fixed by attaching a thin PVC cover over the central polystyrene core to serve as a barrier [34].

At the bone–implant contact, the impact of adding cellular structures also shows enormous potential. The cellular structure can be further treated with a biomaterial to unlock a large potential for medicinal applications. High-molecular-weight bone morphogenetic proteins were injected into the titanium cellular structure in an in vivo investigation to promote bone regeneration. The medullary canal and cortex, as well as the whole segmental bone defect, fully recovered after four weeks [35]. They are used to manufacture aircraft wings, fuselages, control surfaces and interior components. Sandwich lightweight construction reduces fuel consumption and increases payload, and it is used in the construction of boats, yachts and ships. Moreover, it provides superior stiffness and strength at a minimal weight for improved performance and fuel efficiency. Sandwich composites are widely used for hulls, decks, bulkheads and superstructures. Cellular solids structures are used in renewable industries such as photovoltaic modules. This is because their lightweight design facilitates installation and transportation, while their structural rigidity protects delicate solar cells. They are even used in electronic devices and cases to provide structural support, thermal management and electromagnetic shielding. This is because they help protect electronic components and ensure proper function. These are a few of the many illustrations of the wide range of applications for sandwich structures. Its light weight, high strength-to-weight proportion and flexibility make it suitable for a wide range of businesses where weight reduction, toughness and execution are key variables. The fabrication of porous CsPbBr3 and H-CTFs nanocomposites (CsPbBr3/CTFs) has been developed for enhanced photocatalytic H_2_O_2_ production due to their homogeneous active sites distribution showing a unique hollow porous framework [36,37].

## 5. Properties of Cellular Structures

**Figure 12:**
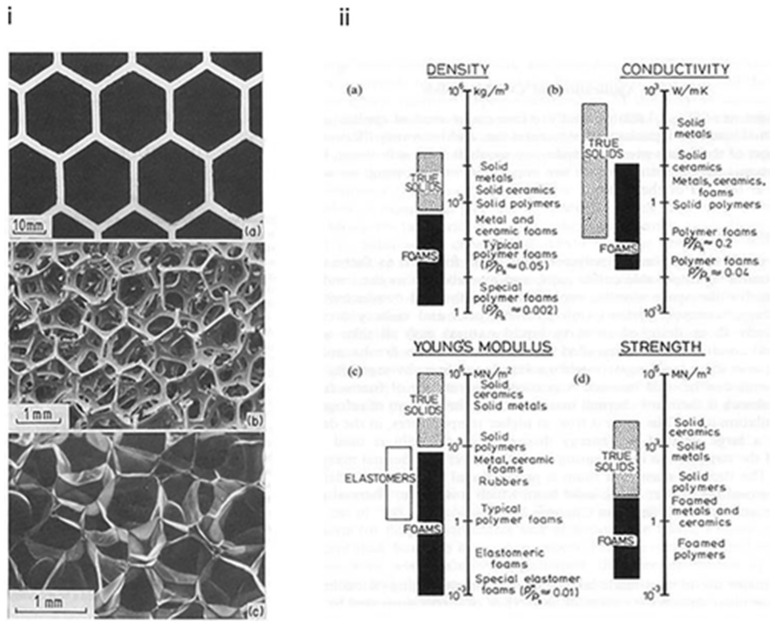
(**i**) Properties of cellular solids: (**a**) a two-dimensional honeycomb; (**b**) a three-dimensional foam with open cells; (**c**) a three-dimensional foam with closed cells. (**ii**) The range of properties available to the engineer through foaming: (**a**) density, (**b**) thermal conductivity, (**c**) Young’s modulus, (**d**) compressive strength [38].

Cellular solid structures have thermal, physical and mechanical properties that are measured using the same process that can be used for fully hard solids. Figure 12 shows the range of four of these properties: density, thermal conductivity, Young’s modulus and compressive strength [38]. The dotted shaded bar shows the range of the conventional solid property; the solid bar shows the extent of this range that is made possible by foaming. This huge extension of properties creates applications for foams that cannot easily be extended to fully dense solids and offers enormous potential for engineering ingenuity. Their low densities allow lightweight and rigid components to be designed, such as sandwich panels and large portable and floating structures of all types. Their low thermal conductivity allows for cheap and reliable thermal insulation, which can only be improved by expensive vacuum-based methods. Their low stiffness makes the foams ideal for a wide range of applications, such as shock absorbers. Their low strengths and large compressive strains make the foams attractive for energy-absorbing applications [38].

Two broad classifications of cellular solids make up cellular structures, which are strong and light in weight. One is open cell, in which linked pathways travel through each individual pore of the foam or scaffold, and the other is closed cell, in which each pore is fully separated from the others [39]. In many real-world applications, cellular materials operate as a cushion and are loaded dynamically. This loading process is frequently accompanied by a high loading strain rate [40]. When a side accident occurs at a speed of 90 km/h, the protective foam in the car-side door panels can be stretched up to 1500 s^−1^. At 40 km/h, the strain rate of the car’s foam energy-absorbing box approaches 200 s^−1^. The mechanical characteristics of the cellular medium cannot be precisely assessed by the static test result and model when the cellular medium is subjected to greater strain and a higher strain rate during dynamic loading [40]. Therefore, it is crucial to look at their mechanical behavior under dynamic loads in high strain rate loading circumstances. Rather than the regularity of its microstructure, a honeycomb’s nodal connection has a significant impact on its macroscopic characteristics [41]. The cause of this is connected to the honeycomb’s behavior in response to macroscopic stresses, which is bending- or stretch-dominated behavior. Different cell material types from both softwoods and hardwoods, both longitudinal and transverse, may be found in the microscopic cellular variety. These also change as the plant grows, giving the earlywood and latewood various textures and proportions. With reference planes for radial, tangential, transverse or cross-sectional directions, wood is anisotropic. It is well acknowledged that tangential and radial surfaces exhibit comparable wettability tendencies [42]. However, it is challenging to adhere cross-sectional wood surfaces; hence; this is often avoided in wood adhesive joint designs. Sometimes, it might be difficult to connect knots because they frequently have cross-sectional surfaces on wood that have longitudinal faces and other peculiarities.

Due to the existence of flaws in the cellular solid structure, like knots and nonparallel grain, the mechanical characteristics of wood are frequently varied. To lessen the impact of such flaws, glue-laminated wood members have recently been created. To create glue-laminated members, thin wood strips from which the flaws have been removed are glued together. These members’ characteristics are more consistent than those of dimensioned lumber. Another benefit of using glulam sections is that they may be bent and expanded to any size [43]. Utilizing wood elements in sandwich structures can also increase their efficiency. Normally, plywood faces are combined with balsa, foam or honeycomb cores. A material’s specific strength is calculated by dividing its strength by its apparent density. It is a crucial metric for determining how strong and light a material is. Ordinary concrete, low-carbon steel and wood (cut against the grain) have specific strengths of 0.012, 0.053 and 0.069, respectively. A material has more strength and is lighter when its specific strength is higher [44]. To increase a building’s height, decrease its structural weight and save project costs, it is crucial to choose materials with high strengths or to increase their specific strengths. Characterizing and modeling the in-plane and out-of-plane mechanical behavior of honeycomb structures is one of the key challenges in material science. Three regimes, the linear elastic, plateau and densification regions, describe the stress–strain curve for the in-plane deformation process. Due to its high surface-to-volume ratio, surface effects should be considered if the honeycomb structure is nanoscale [45]. Since nanowires or nanorods have the potential to be used in nanodevice applications, the surface effect’s impact on the linear elastic behaviors of nanowires has been the subject of many studies.

## 6. Conclusions

The broad and interesting world of cellular-structured solid materials has been explored in detail in this thorough overview, along with their distinctive features and diverse range of applications. The principles regulating these materials have been expanded on by carefully analyzing their structural traits, mechanical properties, thermal behavior and functional aspects. Cellular-structured solids are adaptable candidates for a wide range of applications across many sectors because of their unique characteristics, which include their low density, high strength-to-weight ratio, good thermal insulation and adjustable porosity. The potential for their innovation and improvement is endless, ranging from medicinal gadgets and sustainable building to aerospace, construction, biomedical and automotive engineering. Looking at the future, cellular-structured solid materials will undoubtedly play a crucial role in directing technological advancements and addressing some of the most pressing issues of our time, such as energy efficiency, environmental sustainability and sophisticated manufacturing. Researchers and designers will tackle the uncommon characteristics of these materials to make notable adjustments that benefit us further and support a more manageable climate. This analysis concludes by highlighting the present state of understanding about cellular-structured solid materials as well as the promising future directions. This is proof of the extraordinary potential of these materials and the limitless ingenuity of scientists and engineers who consistently push the envelope of what is feasible.

## Data Availability

Not applicable.
